# Medication Overdoses at a Public Emergency Department in Santiago, Chile

**DOI:** 10.5811/westjem.2015.11.26068

**Published:** 2016-01-12

**Authors:** Pablo Aguilera, Marcela Garrido, Eli Lessard, Julian Swanson, William K. Mallon, Fernando Saldias, Carlos Basaure, Barbara Lara, Stuart P. Swadron

**Affiliations:** * Facultad de Medicina, Pontificia Universidad Católica de Chile, Programa de Medicina de Urgencia, Santiago, Chile; † University of Southern California, Los Angeles, Department of Emergency Medicine, Los Angeles, California

## Abstract

**Introduction:**

While a nationwide poison control registry exists in Chile, reporting to the center is sporadic and happens at the discretion of the treating physician or by patients’ self-report. Moreover, individual hospitals do not monitor accidental or intentional poisoning in a systematic manner. The goal of this study was to identify all cases of intentional medication overdose (MO) that occurred over two years at a large public hospital in Santiago, Chile, and examine its epidemiologic profile.

**Methods:**

This study is a retrospective, explicit chart review conducted at Hospital Sótero del Rio from July 2008 until June 2010. We included all cases of identified intentional MO. Alcohol and recreational drugs were included only when they were ingested with other medications.

**Results:**

We identified 1,557 cases of intentional MO and analyzed a total of 1,197 cases, corresponding to 0.51% of all emergency department (ED) presentations between July 2008 and June 2010. The median patient age was 25 years. The majority was female (67.6%). Two peaks were identified, corresponding to the spring of each year sampled. The rate of hospital admission was 22.2%. Benzodiazepines, selective serotonin reuptake inhibitors, and tricyclic antidepressants (TCA) were the causative agents most commonly found, comprising 1,044 (87.2%) of all analyzed cases. Acetaminophen was involved in 81 (6.8%) cases. More than one active substance was involved in 35% of cases. In 7.3% there was ethanol co-ingestion and in 1.0% co-ingestion of some other recreational drug (primarily cocaine). Of 1,557 cases, six (0.39%) patients died. TCA were involved in two of these deaths.

**Conclusion:**

Similar to other developed and developing nations, intentional MO accounts for a significant number of ED presentations in Chile. Chile is unique in the region, however, in that its spectrum of intentional overdoses includes an excess burden of tricyclic antidepressant and benzodiazepine overdoses, a relatively low rate of alcohol and recreational drug co-ingestion, and a relatively low rate of acetaminophen ingestion.

## INTRODUCTION

A medication overdose (MO) is defined as the ingestion of a medication in an amount that exceeds recommended dosages.^1^ Intentional MOs are an important problem in the emergency department (ED) due to their potential lethality, related hospital costs, and association with mental illness. Overdose is the most common form of suicidal behavior treated in hospital, accounting for 1% of all admissions.^2^–^3^ Knowledge of local patterns of MO is critical for emergency physicians as they attempt to quickly identify and appropriately manage life-threatening overdoses. It is also important that public health officials have the data necessary to direct policy, target interventions and appropriately allocate resources.

The incidence of suicide is increasing around the world, and Chile is no exception. Suicide rates have risen from 4.8/100,000 in 1992 to 12.7/100,000 in 2009.^4^ Moreover, it is estimated that for every successful suicide there are 10 failed attempts. According to the World Health Organization (WHO), suicide by MO is a major public health problem worldwide.^5^ The most common medications used in intentional MO are acetaminophen, benzodiazepines, and tricyclic antidepressants (TCA).^6^

Most of the current Chilean epidemiological data regarding intentional and accidental MO are provided by the Centro de Información de la Pontificia Universidad Católica de Chile (CITUC). CITUC was created in 1992, and has been the national poison control telephone referral center since 2009. CITUC can be accessed by both the lay public as well as by health professionals for guidance in the management of toxic ingestions and exposures via a toll-free number. According to CITUC, 49% of all calls from 1995 to 2002 were related to some type of MO.^7^ MO constituted 41% of all non-intentional poisonings and 88% of all intentional poisonings in 2004; domestic and industrial pesticides and other chemicals accounted for the other causes of intentional poisonings. In 2010, an increase in MO-related calls (58.4%) was noted as compared to 49% between 1999 and 2002.^8^ It has been reported from CITUC data that approximately half of the calls regarding MO involved treatment in an ED.^7.8^ To date, there is no required reporting of MO events by treating physicians.

Hospital Sótero del Río (HSDR) is a public teaching hospital in Chile that serves the southeast population of metropolitan Santiago. This tertiary care hospital has 779 beds and an extremely busy ED, with over 150,000 patient visits per year. It is also the primary provider of emergency services for this diverse population, including emergency services for pediatric and obstetrical/gynecologic patients. HSDR serves both suburban and rural communities, providing care for a catchment area that includes nearly 1.5 million, or roughly 10% of the entire population of Chile.^10^ Within this population, 7.1% are 65 years of age and older (compared to 9.0% nationally), and 22.9% are less than 15 years old (compared to 22.3% nationally). Females comprise 50.6% (compared to 50.5% nationally). Estimates of the poverty rate in the served area (using the WHO standard) range from 9.1% to 17.2% (compared to 14.4% nationally) and the rate of indigence from 1.6% to 4.6% (compared to 2.8% nationally). The hospital mortality rate is 3.7%, which is less than the national mean of 5.2%.^11^,^12^

In this study, we attempted to better characterize the spectrum of intentional MO and its true burden at one of Chile’s busiest public hospitals. These data are complementary to previous studies published on MOs using the CITUC database, which captures only cases that are initiated by a phone call to the Center.

## METHODS

After approval by the institutional research and ethics board, we performed an explicit chart review of patients presenting to the ED at HSDR. We conducted our chart review based on the framework suggested by Kaji et al.^9^

### Search and Chart Review Methodology

A very basic electronic database exists, which includes the following (admittedly limited) information regarding all patient visits in the HSDR ED: chief complaint, medical record number, and basic demographic data. This system is separate from the ED’s paper records and electronic ordering, as well as the hospital’s inpatient paper and electronic medical record. We searched the electronic database for the terms “medication intoxication,” “overdose,” “drug ingestion,” “poisoning,” “suicide” and “intent to commit suicide” in the chief complaint. This search provided several thousand patient ID numbers. All records that indicated a probable MO were selected for further examination. We reviewed hand-written ED and electronic inpatient records to determine which patients were subsequently admitted to the hospital. For this initial screening no charts were excluded based on age or any other epidemiologic patient characteristics (Figure).

These charts were then retrieved and reviewed by the authors. Abstracted data included date of visit, gender, age, origin, pulse, blood pressure, type of pharmaceutical ingested (if known), any street drugs or alcohol use, mortality, disposition, and hospital length of stay (for admitted patients). When intent was specifically documented, it was noted. Laboratory results were generally unavailable as they represent a prohibitive cost in Chile. Therefore, determination of medication type, amount, and co-ingestions was based on the history provided. As details regarding interventions were generally unavailable, patients who presented with abnormal vital signs were identified. These included a pulse below 50 or over 120 beats/min and/or a systolic blood pressure below 90 mmHg that would have likely required (and presumably received) resuscitative measures. We were not able to select patients based on respiratory rate, oxygen saturation of temperature because those vital signs were not registered in the initial triage chart. The drugs ingested were identified when possible and compared with national and international data. As data were recorded directly from charts and no data interpretation was required, we did not calculate a Kappa score.

If any component of a patient chart was illegible, missing, or left blank, that specific data was considered missing, but the rest of the information was used for selected frequency analysis. In order to exclude accidental pediatric ingestions, we included only charts of patients eight years of age and older in the analysis.^14^ We then analyzed the data using Stata 9.0; means, medians, percentages and odds ratios with 95% CI and p values were calculated and reported as appropriate.

## RESULTS

We identified a total of 1,557 patient records, representing 0.51% of all ED presentations between July 2008 and June 2010. The total ED census over this period was 305,294 patient visits. This represents a mean of approximately 2.1 MO patients per each 24-hour period. Females represented 67.6%. Mean age was 28 years (SD=13), with a median of 25 years, and patient ages ranged from 8–89 years. The frequency of MO by age is summarized in Table 1. A peak incidence of MO was observed at age 15.

Of the entire cohort, 81 patient visits had complete missing records and a further 63 left without being seen, therefore providing no information beyond the presenting complaint of MO. Of the 1,413 remaining patient visits, 216 suffered from MO of unknown pharmaceuticals or had charts that were illegible to the point that no medication(s) could be identified. Thus, for a total of 360 (23.1%) patient visits, no specific information regarding pharmaceutical class was available (Figure). The remaining 1,197 (76.8%) had one or more medications documented. Thirty-five percent of these MOs (n=545) involved more than one substance. Co-ingestion of alcohol and/or street drugs was recorded in 8.3% of cases. Four hundred and thirty patients (27.6%) were referred from primary care clinics, 169 (10.9%) presented with vital sign abnormalities that would require resuscitation and 345 (22.2%) were admitted. The proportion of ED visits resulting in admission was higher (28.6%) when they were referred from primary care services (Table 1).

When comparing main medication groups, the four most commonly identified active substances were benzodiazepines, with 547 ingestions (45.7%), followed by selective serotonin reuptake inhibitors (SSRIs) (21.3%, n=255), TCA (13.2%, n=158), and acetaminophen (6.7%, n=81). Table 2 shows the percentage of visits with abnormal vital signs that presumably required resuscitation or resulted in admission to the hospital.

Table 3 lists the top 10 active substances involved (representing 96.9% of all presenting cases of MO). Clonazepam was the most commonly ingested medication, while amitriptyline was associated with the greatest number of vital sign abnormalities on presentation. Admission rates were higher when carbamazepine and amitriptyline were involved. Acetaminophen was associated with the longest length of stay. The youngest mean age at presentation was found with acetaminophen.

We noted seasonal differences, with a higher number of ED visits for MO in the spring (31.5%, n=491) and the lowest incidence during the winter (20.2%, n=315). (p<0.05)

Six patients (0.39%) of the 1,557 died during hospital stay. No patients expired during their stay in the ED.

## DISCUSSION

These results in our population in metropolitan Santiago are overall consistent with previously published studies of the Chilean population. Moreover, we present new data that provide a level of clinical detail not previously reported, including blood pressure and heart rate abnormalities as a surrogate for severity on initial presentation, admission rates, hospital length of stay, and mortality. None of these data were reported in previous studies. Our findings generally confirmed both a 10-year CITUC report from 1992–2002 and another CITUC report in 2004.^7^,^8^ The prior CITUC studies represent a larger proportion of the country over a longer period of time. However, the database comes from self-reports by physician phone calls or from family member phone calls and may not be representative of all MO events. Ultimately, the data from our study and prior UC studies are complementary and should be used together to better understand the needs of the population they describe.

The prevalence of drug classes such as muscle relaxants and NSAIDS were noted to be the second and third most common drugs in intentional overdose in our study. This confirms previous findings in the CITUC studies. Similar to reports from other countries (including our own), we found a strong female predominance (67.5%).^16^,^17^

It is concerning that the peak incidence of MO in our study was 15 years of age, which is at younger than what was previously reported in the Chilean literature.^15^ It is unclear whether this represents a trend of younger MO overdoses or the differences between the populations studied (nationwide call center data versus our metropolitan institution’s patient population).

The results of our study diverged from those of other countries in some key ways. For example, per the American Association of Poison Control Centers, analgesics were the main cause of human exposure calls in North America.^15^ In our population, we found a predominance of MO involving drugs with action on the central nervous system (benzodiazepines and antidepressants).

In Chile, the reported prevalence of depressive symptoms was recently reported at 25.7% for women and up to 8.5% for men.^18^ These numbers are markedly higher than those reported in the United States where the average prevalence is 6.7%.^18^,^19^ There are also more cases of MO involving medications used to treat nervous system diseases in Chile than in the U.S.^15^ A 2003 study found that 62% of all medications administered in Chile that year were related to the central nervous system.^20^ This correlates with the incidence of MO in our study.

Most importantly, we found that the combined prevalence, morbidity, and mortality of TCA were greater than other agents. Patients who ingested TCA were more likely to present with vital signs abnormalities, and thus likely required resuscitation. Specifically, vital sign instability that would require resuscitation was seen in over 20% of cases, compared with less than 10% in patients who ingested benzodiazepines (OR 1.84, 95% CI [1.18–2.85]). TCA overdose had an associated rate of admission to the hospital over twice that of other medications. Furthermore, despite constituting less than 10% of presenting cases, the mortality in these cases represented one-third of all deaths.^2^,^6^ The excess burden of TCA in our study may be related to its availability without a physician prescription until December 2009.

Co-ingestion with alcohol and illicit drugs, primarily cocaine, occurred less frequently in our population than reported in studies in other countries.^18.19^ Furthermore, acetaminophen overdose is not as large a public health problem in Chile as it is in the U.S.^15^ or the UK^22^ in terms of case numbers, but the costs associated with admission and increased length of stay were noted in the present study.

In other countries, such as Brazil, hospital-based systems have been put in place to monitor not only the adverse effects of medications but also the rates of inappropriate use, including cases of MO.^23^ In Chile, however, no such system is in place, and therefore, despite its limitations, our study provides the most representative epidemiological data to date. It has become apparent that further work needs to be done to ensure accurate representation of MO, including the classifications, interventions, adverse events, and outcomes. Despite shortcomings, our large sample size drawn from a hospital serving 10% of the Chilean population is likely representative of the true burden of MO and provides outcome data not previously available.

Our study underscores the need for a nationwide hospital-based pharmaceutical overdose monitoring system (similar to the one in place in Brazil) that could provide this kind of data more contemporaneously without the need for time-consuming retrospective chart review. This would enable Chile’s emergency practitioners to better understand the epidemiological trends in their local patient populations and stay ahead of emerging developments in prescription drug abuse. This information is also important for public health officials so that they may lead efforts to promote public safety and harm reduction.

## LIMITATIONS

This study has a number of important limitations related to the medical records system at HSDR. The ability to identify subjects was dependent on the initial classification of the intake nurse or emergency practitioner in the ED. Many charts were missing, incomplete, or illegible. Patients who did not present with a known or suspected overdose were unavailable for inclusion in the study. Some cases of pharmaceutical intoxication may have been overlooked, in particular, acetaminophen, which may present without symptoms. It is impossible to know to what extent this occurred. Moreover, it is also likely that some patients with MO were misdiagnosed and therefore not included. Furthermore, these data were inherently limited to cases of intoxication leading to ED presentation. Patients not transported to the hospital, such as completed suicide or a self-limited intoxication, would not have been included. Finally, the lack of an available toxicological laboratory made laboratory confirmation impossible. In general, these factors likely led to a systematic underestimation of the true burden of MO in Chile.

This study was also hindered by the lack of completeness of the written ED record and the electronic inpatient record. The completed intervention performed on these patients was not always able to be extracted from the records and was thus not included in this study. To address this issue, we included the proxy measure of abnormal vital signs on presentation that in turn only included blood pressure and heart rate, as the rest of vital signs were not systematically recorded. This likely resulted in underestimating the number of patients requiring intervention, as a patient who has suffered MO may have normal vital signs (and thus not be captured by this measure).

## CONCLUSION

It is clear that MO is a significant problem in Chile. The leading causes of intentional overdoses of patients who seek medical care in the ED include an excess burden of TCA and benzodiazepine overdoses, a relatively low rate of alcohol and recreational drug co-ingestion, and a relatively low rate of acetaminophen ingestion as compared to what has been reported in the international literature.

There remains a great need for a more robust and complete system for data collection to advance the care of patients with MO in Chile.

## Figures and Tables

**Figure:**
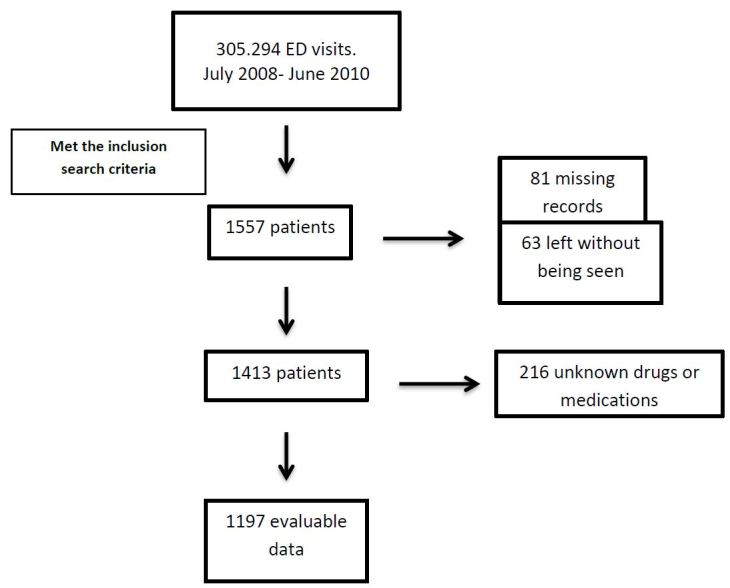
Flow diagram of the study chart review. *ED,* emergency department

**Table 1:** Demographic characteristics of patients presenting with medication overdose to the emergency department at Hospital Sótero del Río, Santiago, Chile.

Characteristics	Category	N	Percentage (%)
Gender	Female	1,052	67.6
Age range (years)	<18	387	24.9
	18–64	1,156	74.2
	≥65	14	0.9
Arrival	Referred from PC services	430	27.6
	Direct ED presentation	1,127	72.4
Abnormal VS potentially requiring resuscitation*	Abnormal VS	169	10.9
	Normal VS	1388	89.1
Active substance identified	One active substance	652	41.9
	>1 active substance	545	35.0
	Not identified	360	23.1
Co-ingestion with alcohol	Yes	114	7.3
	No	1443	92.7
Co-ingestion with street drugs (principally cocaine)	Yes	15	1,0
	No	1542	99.0
Disposition	Home	1149	73.8
	Admitted	345	22.2
	Missing data	63	4.0
Mortality	Deaths	6	0.39

*PC,* primary care; *VS,* vital signs; *ED,* emergency department

**Table 2:** Main medication categories and patients presenting with vital-sign instability after medication overdose to the emergency department at Hospital Sótero del Río. Chile (out of 1197 patients).

Medication group	At least one of these active substances n (%)	Vital sign abnormality requiring resuscitation OR (CI)**	p	Admission OR (CI)**	p
Benzodiazepines	547 (45.7)	0.46 (0.32–0.67)	<0.05	0.25 (0.19–0.33)	<0.05
SSRIs*	255 (21.3)	0.88 (0.57–1.35)	0.543	0.6 (0.43–0.85)	0.002
Tricyclic antidepressant	158 (13.2)	1.84 (1.18–2.85)	<0.05	2.28 (1.59–3.25)	<0.05
Acetaminophen	81 (6.77)	0.75 (0.34–1.58)	0.0421	3.39 (2.32–6.5)	<0.05

*SSRIs,* selective serotonin reuptake inhibitors

**Table 3:** Top 10 active substances identified (n=1197).

Identified substances	Patient rate (%)	Absolute number of exposures	Age average (range)	Transferred from PC (n)	Hospital admission (n)	Mean stay in days
Clonazepam	26.5	317	30.9 (11–62)	67	46	3.24
Amitriptyline	12.5	150	30 (9–68)	64	68	2.45
Alprazolam	9.7	116	32.1 (11–85)	20	16	3.38
Fluoxetine	9.4	113	24.3 (11–50)	29	24	4.26
Sertraline	8.8	105	26.8 (12–59)	31	21	2.19
Diazepam	7.4	89	30.6 (8–63)	22	11	2.82
Acetaminophen	6.7	81	16 (14–54)	30	29	5.62
Cyclobenzaprine	6.1	73	27.1 (9–50)	25	18	1.83
Carbamazepine	5.1	61	25.4 (13–55)	30	31	4.56
Zopiclone	4.7	55	31.7 (8–65)	12	5	2.75
Total*	97	1160	27.5 (11–85)	330	269	3.15

*PC,* primary care

* Top 10 overdoses comprised 96.6% of all cases.
